# Supplementary material for: The interactome of intact mitochondria by cross-linking mass spectrometry provides evidence for coexisting respiratory supercomplexes
Source: Mol Cell Proteomics. 2017 Dec 8;17(2):216–32. doi: 10.1074/mcp.RA117.000470 (PMC5795388; doi:10.1074/mcp.RA117.000470)
Supplement: Supplemental Data [file supp_RA117.000470_133922_0_supp_23978_fzffwf.zip › spectra_annotation/mito_spectra_annotation/113-1-1-1-13-1.pdf]

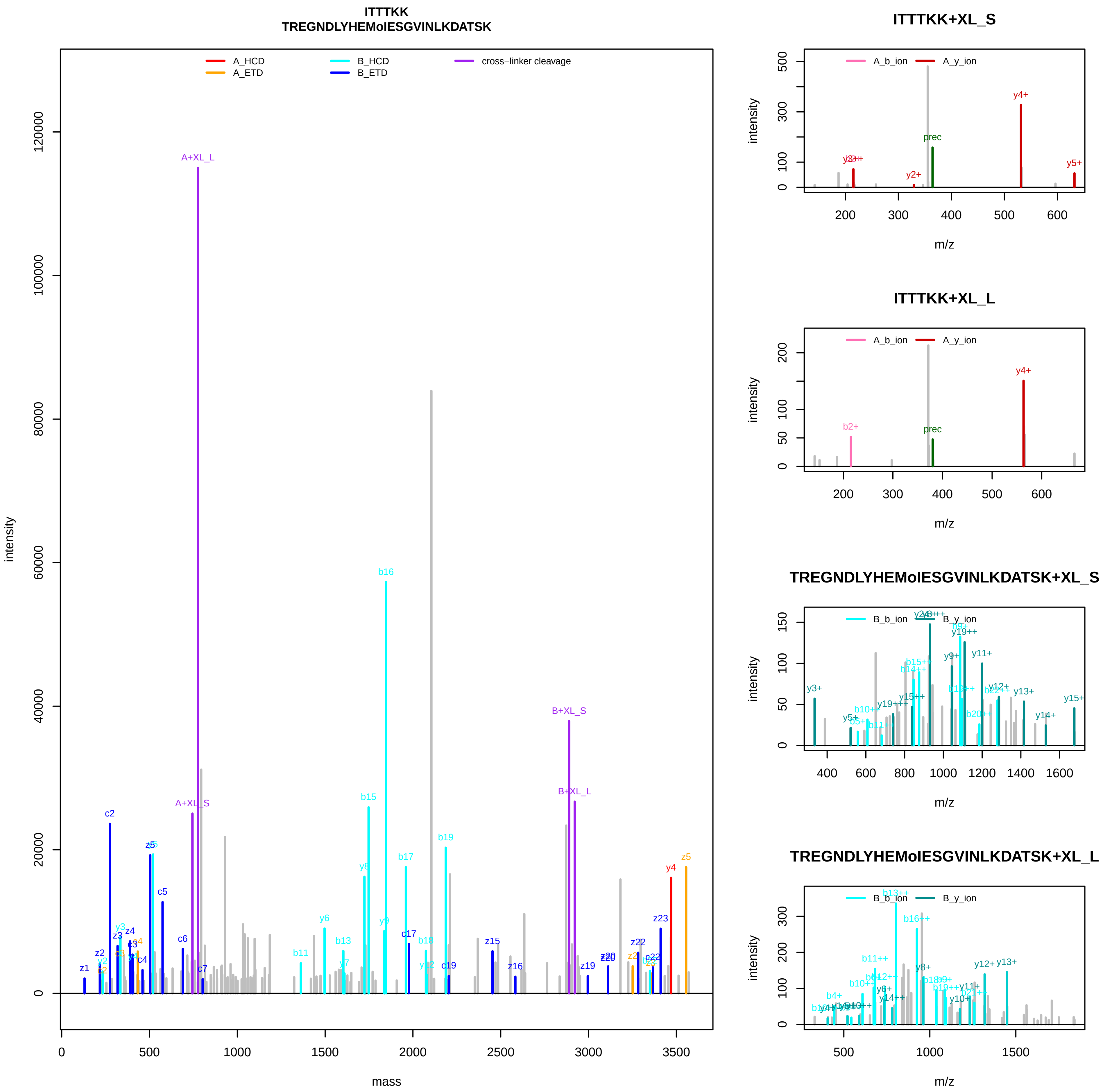

ITTTKK
TREGNDLYHEMoIESGVINLKDATSK
A_HCD
A_ETD
B_HCD
B_ETD
cross−linker cleavage
A+XL_L
A+XL_S
B+XL_S
B+XL_L
b16
b15
b17
b19
y4
z5
c2
intensity
mass
ITTTKK+XL_S
A_b_ion
A_y_ion
y4+
y5+
prec
y2+
m/z
ITTTKK+XL_L
b2+
TREGNDLYHEMoIESGVINLKDATSK+XL_S
B_b_ion
B_y_ion
y3+
y11+
y13+
y15+
y14+
TREGNDLYHEMoIESGVINLKDATSK+XL_L
b16++
y13+
y12+
